# Risk of Potentially Neurotoxic Exposure in Infants Under High-Dose Cefepime Treatment—A Pharmacometric Simulation Study

**DOI:** 10.3390/pharmaceutics17050544

**Published:** 2025-04-22

**Authors:** Verena Gotta, Chantal Csajka, Antonia Glauser, Christoph Berger, Marc Pfister, Paolo Paioni

**Affiliations:** 1 Pediatric Pharmacology and Pharmacometrics, University of Basel Children’s Hospital, 4031 Basel, Switzerland; 2 Pediatric Clinical Pharmacy, University of Basel Children’s Hospital, 4031 Basel, Switzerland; 3 Center for Research and Innovation, University Hospital and University of Lausanne, 1011 Lausanne, Switzerland; 4 School of Pharmaceutical Sciences, University of Geneva and University of Lausanne, 1211 Geneva, Switzerland; 5 SwissPedDose, 8008 Zurich, Switzerland; 6 Division of Infectious Diseases, University Children’s Hospital Zurich, 8008 Zurich, Switzerland

**Keywords:** cefepime, neurotoxicity, infants, neonates, children, pharmacokinetics, pharmacodynamics, precision dosing, model-informed dosing, pharmacometrics

## Abstract

**Background:** Optimal dosing of cefepime in infants 1–2 months remains undefined. **Objectives**: We aimed to quantify the risk of potentially neurotoxic exposure with high-dose cefepime (50 mg/kg/8 h) in infants 1–2 months of age, as compared to adjacent age groups (neonates, infants 2–12 months) and lower dose treatment (50 mg/kg/12 h). **Methods**: Pharmacometric simulations were performed using two published population pharmacokinetic models combined with demographic data, including serum creatinine, for neonates and infants ≤ 12 months. Adult-derived safety thresholds for potential neurotoxicity were defined as steady-state trough concentration (C_trough_) > 20 or > 35 mg/L, respectively. The corresponding probability of target attainment (PTA) was calculated as free concentration, 50% of the time during the dosing interval above the minimal inhibitory concentration (MIC) breakpoint of 8 mg/L (*Pseudomonas* spp.) (50% fT>MIC_8mg/L_). **Results**: The predicted risk of C_trough_ > 20 (>35) mg/L under high-dose cefepime was 40–54% (12–22%) in infants 1–2 months while providing high PTA (100%). It was predicted to be 1.3–1.7 fold higher in neonates (model 1), and reduced 1.8–2.4 fold in infants 2–12 months (model 1), or to be similar (model 2), respectively. Both models predicted approximately 2–4 fold reduced risk using lower dose treatments while maintaining high PTA (≥97%). **Conclusions**: The risk of potential neurotoxic concentrations in infants > 1 month treated with cefepime 50 mg/kg/8 h is high if defined by adult safety thresholds. Lower dose cefepime in infants 1–2 months could be a safe option without compromising PTA, if defined as 50% fT>MIC_8mg/L_. Achievement of 100% fT>MIC_8mg/L_ may require prolonged infusion time even under high-dose treatment. Future research is required to evaluate potentially age-dependent safety thresholds.

## 1. Introduction

Cefepime is a fourth-generation intravenous cephalosporin antibiotic with a structure that allows better and more rapid penetration through the cell wall of aerobic Gram-negative bacteria compared to 3rd generation cephalosporins [1]. It is able to avoid destruction by some beta-lactamases and is active against a broad spectrum of Gram-negative pathogens, including *Pseudomonas aeruginosa.* [1] Thus, cefepime is indicated for the treatment of severe infections, such as febrile neutropenia (FN) [2,3] and, in combination with metronidazole, for complicated intra-abdominal infections in adults [4] and children [5]. For FN, pediatric randomized controlled studies have established equal efficacy compared to alternative antibiotics at doses of 50 mg/kg every 8 h [6,7] or 12 h [8,9]. In general, intravenous application every 8 h is required for infections caused by *Pseudomonas* spp., due to higher MICs [10].

Pharmacokinetic studies in children [11,12,13] and neonates [14,15,16] have shown the importance of renal function maturation for cefepime clearance, justifying lower doses in neonates (30–50 mg/kg every 12 h) [17] compared to infants and children (50 mg/kg every 8–12 h). However, dosing guidelines lack consensus, particularly for infants 1–2 months: dosing uncertainty is acknowledged by the Swiss product label [18], whereas no recommendation is given for infants < 2 months of age in the FDA label. [19] In this age group, normal eGFR (mL/min/1.72 m^2^) amounts to approximately 50–70% of the eGFR of older infants and children [20], which may put them at risk for overexposure and neurotoxicity [17]. Neurotoxicity of cefepime resembles other beta-lactam antibiotics [21] and may manifest clinically with altered mental status, myoclonus, seizures, and/or aphasia [22]. These symptoms are thought to be mediated by antagonistic binding to gamma-aminobutyric acid (GABA) receptors, reducing GABA-mediated inhibition of excitatory neurotransmitter signals [23].

The dose-dependent risk of overexposure in infants 1–2 months of age as compared to adjacent age groups (neonates and infants > 2 months of age) receiving standard doses of cefepime has not yet been specifically evaluated. A drug concentration of >20 mg/L [22] or >35 mg/L [1], respectively, has been proposed as a “safety threshold” for increased risk of neurotoxicity in adults. Data on pediatric patients are scarce [24,25], as detection of neurotoxicity can be challenging [1].

The objective of this pharmacometric study was to enhance precision dosing of cefepime in infants, by leveraging existing data to optimize the risk–benefit evaluation of dosing decisions. More specifically, we aimed to quantify both the risk of elevated, potentially neurotoxic cefepime exposure and the probability of efficacy target attainment (PTA) in infants 1–2 months of age treated with cefepime 50 mg/kg every 8 h (high-dose), as compared with adjacent age groups (i.e., neonates < 1 months of age, infants 2–12 months of age) and different lower dose regimens.

## 2. Materials and Methods

### 2.1. Study Design

A pharmacometric simulation framework was established based on two identified suitable population pharmacokinetic models that characterized the pharmacokinetics of cefepime in infants aged 1–2 months and in adjacent age groups [12,13]. Given that cefepime is primarily eliminated by the kidneys, it was deemed essential to incorporate real-world demographic data reflecting the typical renal function of infants treated for infections. Therefore, the necessary patient demographics for model simulations—including covariate distribution with serum creatinine measurements—were sourced from a previously reported population of neonates and infants (i.e., 0–12 months of age) who were receiving gentamicin [26].

### 2.2. Pharmacometric Models

Following an initial review of available pharmacokinetic data for cefepime in neonates, infants, and pediatric patients [11,17,27,28,29], five published population pharmacokinetic models were identified from the literature [12,13,14,15,16]. Out of the five potential pharmacometric models, two were selected as the most suitable for exposure simulations in this virtual study, as they included infants 1–2 months of age and older in their development, along with relevant covariate analysis (weight, renal function) and reported model evaluation metrics (e.g., bootstrap, simulation-based diagnostics like prediction corrected normalized predictive check) [12,13]. A flow diagram illustrating model selection is presented in Supplemental Figure S1.

Both pharmacometric models describe cefepime clearance as a non-linear function of body weight through allometric scaling and renal function, which is quantified either by serum creatinine or eGFR. The model from Shoji et al. additionally includes a maturation function according to postmenstrual age (PMA) [12]. Both models describe the volume of distribution as a linear function of weight, [12,13] and the model from Shoji et al. additionally incorporates the influence of gestational age (GA) [12].

#### 2.2.1. Pharmacometric Model 1: Neonates, Infants, and Children

Shoji et al. (2016) [12] describe the combined cefepime pharmacokinetics of two previously reported populations of 54 neonates [16] and 37 infants and children aged 2 months–16 years [11]. In both studies, a prospective serial/rich sampling approach was chosen (2–8 cefepime samples obtained during the dosing interval per patient at pre-defined time points). The best model fit was achieved using a 2-compartment model to describe the distribution kinetics. The typical clearance (CL) was described as a function of weight (in kg, standard allometric scaling), serum creatinine (SCr, in mg/dL), and postmenstrual age (PMA, in weeks, maturation function):

CL (L/h) = 0.395 weight^0.75^ × (SCr/0.6)^−0.392^ × [−0.09 + 1.09 × (1 − exp^(−0.00958 × PMA)^)] (1)

The typical volume of distribution at steady-state (Vss) was described as a function of weight (in kg, standard allometric scaling) and gestational age (GA, in weeks) as follows:

Vss (L) = 0.406 × weight × (GA/30)^−0.548^ (2)

Inter-compartmental clearance (Q) was estimated to be 0.575 L/h × weight^0.75^, the central volume of distribution (V1) to 0.46 of Vss. Unexplained inter-individual variability was quantified to 32% in CL and 22% in the volume of distribution (assumed to apply to Vss), and residual variability was 66.3%.

#### 2.2.2. Pharmacometric Model 2: Critically Ill Infants and Children

De Cacqueray et al. (2022) [13] describe cefepime pharmacokinetics in a population of 59 critically ill pediatric patients, 1.1 months–17.6 years, mainly with lung or bloodstream infections (inclusion criteria: weight > 3 kg). In total, 129 plasma concentrations were collected (timing not reported). A one-compartmental distribution model was developed, also including standard weight-based allometric scaling for clearance and volume of distribution (V); estimated glomerular filtration rate (eGFR, in mL/min/1.73 m^2^, estimated by Schwartz [30]) was included as a covariate on clearance (CL):

CL (L/h) = 1.21 × (weight/9)^0.75^ × (eGFR/153)^0.37^ (3)

V (L) = 4.8 × (weight/9)(4)

The remaining unexplained inter-individual variability was 39% in CL and 35% in V. Residual intra-individual variability was 39%.

### 2.3. Virtual Patient Population

Demographic patient data required as covariates for pharmacometric simulations were taken from a previously reported prospective pharmacokinetic study in pediatric patients aged 1 day to 10 years receiving gentamicin [26]. The study was approved by the Ethics Committee, Zurich, Switzerland (BASEC 2017-01296). The study population was enriched with demographic data of patients treated with gentamicin during the same time period, not participating in the prospective study but agreeing in writing on data reuse. For the reuse in the current study, the data were fully anonymized. GA was stratified into three groups: extremely preterm (<28 weeks), very preterm (28 to <32 weeks), and moderate to late preterm (32 to <37 weeks). Only patients ≤12 months were included in this simulation study, stratified into 3 main populations: neonates < 28 days, infants 1–2 months (28–59 days), and infants 2–12 months (60–365 days) of age. A further subpopulation included term neonates (GA > 36 weeks). Serum creatinine values reported as <27 µmol/L (occurring in 137 (58%) of patients) were imputed by the value of 27 µmol/L. Non-plausible age (<0 days) and gestational age (0 weeks) were treated as missing. One patient with implausible weight for age (>10 kg neonate) was excluded. According to the pharmacometric models applied, the estimated glomerular filtration rate (eGFR) was calculated according to two Schwartz equations developed for children [30] and full-term infants [31] for the sensitivity analysis described below, respectively. The primary analysis was performed with these measured/calculated surrogates of renal function.

### 2.4. Cefepime Exposure Simulations and Outcomes

#### 2.4.1. Dose-Exposure Simulations

Exposure simulations for the first four dose applications were conducted using the software Simulx (Version 2021R2, Lixoft, France). From each model individual concentration-time profiles from 500 patients (randomly sampled from each defined subpopulation) were generated by Monte Carlo simulations, given the dose (50 mg/kg), dosing interval (8 h, and 12 h in neonates with GA > 36 weeks as well as infants 1–2 months), covariates (weight, serum creatinine, eGFR, PMA and GA) and between-subject variability in cefepime clearance and volume of distribution. Statistics and figures were created using R (R version 4.2.1, R Foundation for Statistical Computing, Vienna, Austria).

#### 2.4.2. Safety- and Efficacy-Related Exposure Outcomes

Safety and efficacy target attainment was evaluated at steady-state. A steady state was assumed after 4 doses (24 h–32 h), consistent with cefepim’s half-life of 3.6 ± 0.6 h reported in term infants [12].

*Safety-related exposure outcome*. As a safety-related exposure outcome, potentially neurotoxic total C_trough_ was defined as >20 or >35 mg/L, i.e., derived from clinical data in adults [1,22]. The individual predicted C_trough_ at the end of the dosing interval was extracted, and the percentage (%) of patients with total C_trough_ > 20 mg/L or >35 mg/L was calculated for each subpopulation and model.

*Efficacy-related exposure outcome.* For *Pseudomonas* spp., a minimal inhibitory concentration (MIC) breakpoint of 8 mg/L or higher is classified as resistant to Cefepime, while for other pathogens, the resistance breakpoint is at 4 mg/L [10]. As a beta-lactam antibiotic, the time during which the free drug concentration (fT) remains above the MIC (fT>MIC) is considered relevant for efficacy. The exact pharmacokinetic–pharmacodynamic (PK-PD) target is not clearly defined: preclinical studies suggest 40–70% fT>MIC as a suitable target, whereas in the clinical setting, a target above 70% and up to 100% fT>MIC may provide additional benefit [1]. For the therapeutic drug monitoring of cephalosporins, the target of 45–100% fT>MIC is generally proposed [32].

In this study, the efficacy-related exposure target was defined as the free drug concentration—calculated as 80% of the total concentration (assuming a fraction unbound of 0.8)—remaining above MIC breakpoints of (4-)8 mg/L during 50% (and up to 70–100%) of the dosing interval. Specifically, this target was defined as 50% fT>MIC_8mg/L_ and 50% fT>MIC_4mg/L_. The probability of target attainment (PTA) was defined as the percentage (%) of patients achieving the efficacy-related exposure target.

### 2.5. Sensitivity Analyses

Sensitivity analyses were performed with respect to various factors: (A) dosing, specifically 30 mg/kg every 8 h; (B) serum creatinine and kidney function, using mean eGFR/serum creatinine values appropriate for age as described in the subsequent sections, rather than the reported values from the reference population [26]; and (C) by extrapolating another model (“Pharmacometric model 3”), originally developed from neonates [15], to infants 1–2 months (C) (for model description: see Supplemental Materials).

For sensitivity analysis B, the following mean values for eGFR and serum creatinine by age were used: *<1 month*: 41 mL/min/1.73 m^2^ (mean for 1 week of age [20]) and approximately 44 μmol/L (mean for GA 23–42 weeks, day 14–30 [33]); *1–2 months*: 66 mL/min/1.73 m^2^ (mean for 2–8 weeks [20]) and 19.9 μmol/L (mean calculated for 6 weeks by extrapolation of the equation SCr [μmol/L] = exp^(2.98+0.0735×age [in years])^ [34]); *>2–12 months*: 96 mL/min/1.73 m^2^ (mean for >8 weeks to 2 years [20]) and 20 μmol/L (mean for 12 weeks [34]).

## 3. Results

### 3.1. Pharmacometric Models and Virtual Patient Population

A total of 235 patients ≤ 12 months were included in the analysis. This cohort consisted of 131 neonates < 28 days, including 114 term neonates GA > 36 weeks, 73 infants 1–2 months (28–59 days), and 31 infants 2–12 months (60–365 days) of age. Table 1 provides a summary of patient characteristics. The characteristics used as covariates for model simulations are further detailed in Supplemental Figure S2. The pharmacometric model simulation codes used in this study can be found in the Supplemental Materials.

### 3.2. Cefepime Exposure Simulations and Outcomes

#### 3.2.1. Dose-Exposure Simulations

**Figure 1:**
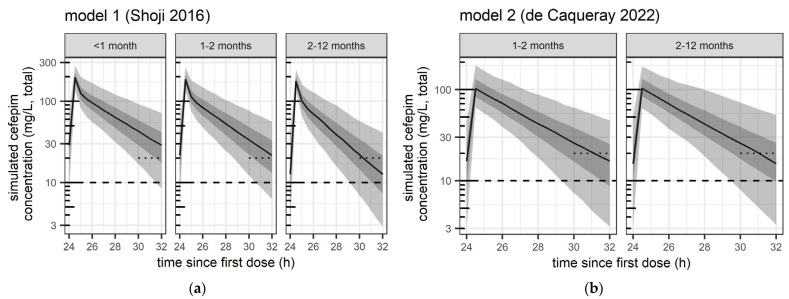
Predicted distribution of exposure from pharmacometric simulations from (**a**) model 1 (Shoji et al. 2016 [12]) and (**b**) model 2 (de Caqueray et al. 2022 [13]),with high-dose cefepime 50 mg/kg every 8 h in all age groups. Safety and efficacy target attainment was evaluated between the fourth and fifth dose (i.e., 24 h–32 h after the first dose), i.e., at steady-state. Black curve: median concentration. Grey shaded areas: 50% prediction interval (25th–75th percentile) and 90% prediction interval (5th–95th percentile), respectively. Dotted short line: trough concentrations > 20 mg/L (evaluated lower safety threshold). Dashed line: Total concentration of 10 mg/L, corresponding to the expected free concentration of 8 mg/L (MIC efficacy threshold evaluated).

The distribution of simulated exposure after the 4th dose (24 h–32 h) from the two models is illustrated in Figure 1 for the main three populations and the evaluated high dose of 50 mg/kg every 8 h.

#### 3.2.2. Safety- and Efficacy-Related Exposure Outcomes

Table 2 summarizes the corresponding proportion of patients with potentially neurotoxic C_trough_ levels (>20 and >35 mg/L) compared to the proportion of patients achieving 50% (and 70–100%) fT>MIC_8 mg/L_ (=PTA). These results are presented for the evaluated high-dose regimen of 50 mg/kg every 8 h (the reference high-dose of pediatric patients) and are compared to a lower dose of 50 mg/every 12 h (=reference high-dose for neonates with GA > 36 weeks).

**Figure 2:**
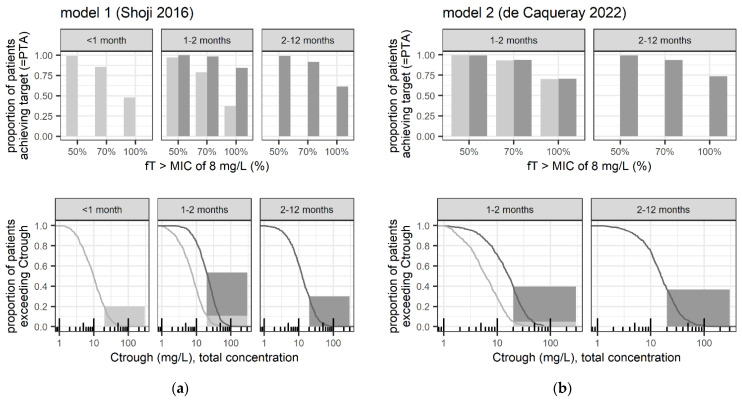
Main simulated safety and efficacy-related exposure outcomes at steady-state according to (**a**) model 1 (Shoji et al. 2016 [12]) and (**b**) model 2 (de Caqueray et al. 2022 [13]), following dosing of 50 mg/kg every 8 h (dark grey lines/bars *=* reference high-dose for pediatric patients) and 50 mg/kg every 12 h (light grey lines/bars *=* reference high-dose for neonates with GA > 36 weeks). **Upper panels**: Evaluated efficacy-related outcome, that is probability of target attainment (PTA) in terms of the proportion of simulated patients with predicted 50% fT>MIC_8mg/L_, and up to 70–100% fT>MIC_8mg/L_. **Lower panels**: Evaluated safety-related outcome, that is proportion of patients with predicted C_trough_ > 20 mg/L, together with the cumulative distribution of simulated C_trough_ (lines) from which proportions for potential alternative safety thresholds can be derived; proportions with C_trough_ > 35 mg/L are given in Table 2.

Figure 2 compares PTA and C_trough_ distribution (including the proportion > 20 mg/L) between high-dose and lower-dose regimens in infants 1–2 months. Additionally, it contrasts these findings with the PTA and C_trough_ distribution (including the proportion > 20 mg/L) observed in adjacent age groups receiving their reference high-dose regimens.

Supplemental Table S1 further summarizes the proportion of patients with efficacy target achievement for high versus lower MIC (fT>MIC_8 mg/L_ versus fT>MIC_4 mg/L_).

### 3.3. Sensitivity Analyses

The proportion of patients with potentially neurotoxic C_trough_ and the proportion of patients achieving 50% (70–100%) fT>MIC_8 mg/L_ under the following conditions are presented in Table 3: (A) a lower dose of 30 mg/kg every 8 h, (B) mean serum creatinine/eGFR adjusted for age, and (C) extrapolation from a neonatal model.

## 4. Discussion

The present study aimed to elucidate the dose- and age-dependent risk of neurotoxic exposure to cefepime in infants, with a specific focus on comparing those aged 1–2 months, who currently lack clear label recommendations, with adjacent age groups. As a virtual study, the work streamlines with the new ICH Guideline E11A for Pediatric Extrapolation (2024), by leveraging existing data to minimize the need for extensive clinical studies in children, and to enhance the risk–benefit evaluation of dosing decisions in children. Our results demonstrate a significant risk of potentially neurotoxic cefepime concentrations in infants under the high-dose regimen of 50 mg/kg every 8 h, if defined by adult thresholds (C_trough_ > 20 mg/L). Even though our analysis using model 1 shows that the risk of neurotoxic concentrations clearly decreases with age, infants aged 1–2 months receiving the high-dose regimen still exhibit a 2.7–4.4 fold increase in the risk of cefepime exposure, exceeding the threshold for potential neurotoxicity compared to newborns treated with the maximal recommended dose regimen of 50 mg/kg every 12 h for this age group (54% versus 20% > 20 mg/L, 22% versus 5% > 35 mg/L) [17]. Additionally, there is a 1.8–2.4-fold increase in risk compared to older infants treated with the same high-dose regimen (54% versus 30% > 20 mg/L, 22% versus 9% > 35 mg/L), suggesting age-dependent vulnerability.

The safety targets evaluated in our analysis were derived from data in adults [1,22], but their relevance to the risk of cefepime-induced neurotoxicity in children remains unclear. Distribution of the drug into the brain may rather be age-independent in the pediatric age range, according to a comprehensive review suggesting a functional blood–barrier already in neonates [35]. However, the developing brain is known to be vulnerable to drugs [35], and information on maturational changes at the blood–brain barrier and regarding pharmacodynamic changes (e.g., age-dependent target receptor expression) is still scarce [36]. Regarding the proposed mechanism of cefepime neurotoxicity, age-dependent pharmacodynamic changes in the antagonistic binding to GABA receptors would be of interest, which is thought to reduce GABA-mediated inhibition of excitatory neurotransmitter signals [23]. Even in adults, exposure above the proposed targets can regularly be expected; yet, it does not necessarily lead to neurotoxicity in all patients. Furthermore, detecting clinical manifestations of cefepime-induced neurotoxicity in young infants might be challenging. Indeed, cefepime-induced neurotoxicity in children is not well documented in the literature. Hambrick et al. [24] summarize six pediatric reports of neurotoxicity, all in patients with chronic kidney disease or acute kidney injury. Only 2 of the reported cases provide information on cefepime plasma concentration, with a trough concentration of 80 mg/L on day 9 of therapy [24] and 62 mg/L five days after the last cefepime dose intake [25], respectively. Figure 2, which illustrates the C_trough_ distribution, indicates that such high C_trough_ levels would only rarely be expected for infants and children with overall normal renal function. Cefepime neurotoxicity rates have been estimated between 4.4 and 23.2% [37,38,39]; pediatric-specific estimates would be desirable. Additionally, variable cerebrospinal fluid/plasma cefepime concentration ratios have been reported [29], with potentially increased ratios in patients with cefepime neurotoxicity [40]. Risk factors for cefepime-induced neurotoxicity include impaired kidney function, particularly end-stage renal disease, due to pharmacokinetic changes and/or increased penetration into the central nervous system (CNS), e.g., mediated by uremic toxins. Other risk factors include older age (with the mean age of patients experiencing neurotoxicity being 65–70 years), inflammatory conditions such as sepsis, critical illness, or severe infections, underlying CNS conditions, and overdose [1,22].

Taking all this information into consideration and based on the predicted trough concentration as a surrogate for neurotoxicity in our analysis, it appears that young infants aged 1–2 months may be at higher risk for cefepime-induced neurotoxicity under the currently recommended dosing regimen for pseudomonal infections (i.e., high dose regimen with 50 mg/kg every 8 h). Although a clearly defined exposure cut-off for the risk of neurotoxicity in children is not available, the data suggest that this age group is particularly vulnerable. This potential risk must be carefully weighed against the predicted efficacy of this regimen. Prospective evaluation and validation of pediatric-specific safety thresholds, e.g., including sensitive electrocardiogram measurements along with cefepime exposure assessments (total and unbound), are strongly recommended to address the discussed knowledge gaps. In our analysis, the predicted PTA was high in all age groups and using all evaluated models, suggesting the effective antibacterial activity of cefepime against *Pseudomonas* spp., as the free drug concentration remained above the MIC breakpoint of 8 mg/L for at least 50% of the dosing interval in ≥99% of simulated patients. If 100% fT>MIC_8mg/L_ is targeted, prolonged or continuous infusion may be required to achieve equally high PTA. This suggests that the dosing regimen of 50 mg/kg every 8 h is likely effective in treating infections caused by pathogens with high MIC values. Yet, most isolates of *P. aeruginosa* in Switzerland have a MIC below 8 mg/L [41], raising the question of whether lower-dose regimens, such as 50 mg/kg every 12 h or 30 mg/kg every 8 h, might be sufficient. In fact, the predicted PTA for the MIC breakpoint of 8 mg/L remained high in infants aged 1–2 months treated with 50 mg/kg every 12 h (≥97% of patients with 50% fT>MIC_8mg/L_, Table 2) or 30 mg/kg every 8 h (≥95% in sensitivity analysis A, Table 3). While the high PTA highlights the effectiveness of cefepime at these dosing regimens, the associated risk of potential neurotoxicity calls for caution and a balanced approach. Clinical practitioners should weigh the benefits of effective antimicrobial therapy against the potential risk of adverse effects. In addition, it must be considered that pathogens other than *Pseudomonas* spp. are mostly characterized by lower MIC breakpoints of ≤4 mg/L [10]. Therapeutic drug monitoring (TDM) could be a valuable tool in optimizing individual patient dosing, ensuring efficacy while minimizing the risk of toxicity, especially in patients with additional risk factors for neurotoxicity, such as impaired renal function or underlying CNS conditions. TDM of cefepime is recommended to safely optimize plasma exposure and clinical outcomes, and may even be cost-effective [42]. As such, a stronger recommendation within clinical guidelines may be advised [3], as well as routine TDM for infants 1–2 months receiving high-dose cefepime, given pharmacokinetic variability. Furthermore, developing pediatric-specific pharmacokinetic–pharmacodynamic models and safety thresholds would enhance the precision of dosing recommendations in this vulnerable population. Prospective clinical studies are necessary to validate these models and to determine the actual incidence of neurotoxicity in pediatric patients treated with cefepime.

Even though our findings offer valuable insights into the pharmacokinetics and potential safety concerns of cefepime administration in young infants, our study has several limitations. First, although a fraction unbound of 80% is assumed to be a reasonable estimate for cefepime [1] and has been used in previous evaluations of pediatric PTA [12,13], actual protein binding may be variable, as suggested by a range of reported unbound fractions of 51.6 to 99.2% [43]. Assuming a higher protein binding would reduce our predicted PTA, and may be expected in neonates and young infants based on limited data from other cephalosporins [44]. Second, residual intra-individual variability was large in both models (66% in model 1 [12] and 39% in model 2, respectively [13]), but cannot be meaningfully incorporated in simulations for evaluation of fT>MIC. This reported substantial variability also suggests that controlling exposure by TDM-based dose adjustment may, in some cases, be challenging, as it indicates potential high variation from one measurement to the other under stable dosing. Third, since approximately half of the serum creatinine values were reported below the limit of quantification of 27 µmol/L, imputing these values as 27 µmol/L in the main analysis may have resulted in an underestimation of actual renal function. Consequently, the predicted percentage of patients exceeding toxicity thresholds may represent a conservative estimate, but a similar age-dependency was suggested in sensitivity analysis B (use of mean serum creatinine/eGFR adjusted for age, Table 3). Future studies may consider incorporating cystatin C for accuracy [34,45]. In children with augmented renal clearance, the risk of potentially neurotoxic exposure might actually be lower, while the PTA may also decrease. Furthermore, uncertainties may remain regarding the serum creatinine assays used in the development of the models applied in this simulation study, and the question about their comparability and adequacy when combined with measurements from our demographic patient population [46]. For this reason, absolute percentages for outcomes may vary depending on the models and assumptions used. However, the relative differences between age groups and dosing regimens are not expected to change significantly for predicted exposure in terms of C_trough_ and fT>MIC. Due to differences in model structure (model 1: a two-compartmental model developed on data including a rich sampling design, model 2: a one-compartmental model developed on sparse data), greater differences in exposure prediction may, however, be expected for peak concentration prediction, which was not investigated as an outcome of interest. For peak concentration prediction, the two-compartmental model predicting higher peak exposure appears preferable (Supplemental Figure S3).

## 5. Conclusions

While a dose of cefepime 50 mg/kg every 8 h is effective against *Pseudomonas* spp. in infants, the potential for neurotoxic exposure remains a significant concern, especially in infants aged 1–2 months. Lower doses (50 mg/kg/12 h or 30 mg/kg/8 h) balance efficacy and safety, but prolonged infusions or TDM may be needed for 100% fT>MIC targets, depending on individual microbiological findings. Future research should focus on evaluating potentially age-dependent safety thresholds in this vulnerable patient population.

## Figures and Tables

**Table 1:** Distribution of patient demographics used in primary analysis. Values are summarized by median (interquartile range), unless otherwise indicated.

**Population**	**<1 Month** **(<28 Days)**	**1–2 Months** **(28–59 Days)**	**2–12 Months** **(60–365 Days)**
Number of patients	131 *	73	31
Age, days	9 [3, 19]	39 [33, 47]	157 [76, 259]
Weight, kg	3.6 [3.1, 3.9]	4.6 [4.1, 4.8]	5.7 [4.7, 6.5]
Height, cm	51.5 [49, 53]	54.6 [53, 56]	62 [57.7, 67.5]
Serum creatinine **, µmol/L	27 [27–44.3]	27 [27]	27 [27–27.5]
Gestational age (GA), weeks	39 [38, 40] *	39 [38, 40]	38 [36, 40]
Postmenstrual age (PMA), weeks	41 [39.1, 42.0]	44.7 [43.4, 46.1]	59.6 [50.8, 72.4]
eGFR, Schwartz 1976 [30], mL/min/1.73 m^2^	87 [56, 95]	97 [91, 101]	108 [97, 117]
eGFR, Schwartz 1984 [31], mL/min/1.73 m^2^	71 [46, 77]	80 [74, 82]	88 [79, 96]
Mean eGFR, KDIGO, mL/min/1.73 m^2^	41 (1 week)	66 (2–8 weeks)	96 (>8 weeks to 2 years)
Mean serum creatinine, μmol/L	44 (day 14–30)	20	20

* including 114 neonates (88%) with GA < 36 weeks. ** values reported as <27 µmol were imputed by this lower limit of quantification of 27 µmol.

**Table 2:** Numerical summary of safety and efficacy-related exposure outcomes at steady-state following dosing of 50 mg/kg every 8 h (=reference high-dose for pediatric patients) or 50 mg/kg every 12 h (=reference high-dose for neonates with GA > 36 weeks), considering a MIC breakpoint of 8 mg/L (MIC_8 mg/L_; see Supplemental Table S1 for MIC_4 mg/L_).

Age	Dosing Regimen	Model 1	Model 2
**Safety: Percentage of patients with predicted C_trough_ > 20(> 35) mg/L**			
<1 month	50 mg/kg every 8 h	68% (38%)	X *
	50 mg/kg every 12 h	20% (5%)	X *
1–2 months	50 mg/kg every 8 h	54% (22%)	40% (12%)
	50 mg/kg every 12 h	11% (2%)	4.8% (0.8%)
2–12 months	50 mg/kg every 8 h	30 (9%)	37% (13%)
**Efficacy: Percentage of patients with predicted 50% fT>MIC_8mg/L_ (Range: 70–100% fT>MIC_8mg/L_)**			
<1 month	50 mg/kg every 8 h	100% (99–92%)	X *
	50 mg/kg every 12 h	99% (85–48%)	X *
1–2 months	50 mg/kg every 8 h	100% (99–85%)	100% (94–71%)
	50 mg/kg every 12 h	97% (79–37%)	100% (93–70%)
2–12 months	50 mg/kg every 8 h	99% (92–61%)	99% (94–74%)

X * Not modeled due to lack of neonatal data in Model 2.

**Table 3:** Summary of sensitivity analyses A–C. A: lower dose of 30 mg/kg every 8 h; B: mean serum creatinine/eGFR for age; C: extrapolation from a neonatal model.

Sensitivity Analysis	A		B		C
	Model 1	Model 2	Model 1	Model 2	Model 3 (Zhao, 2020) [15]
**Safety: Percentage of patients with predicted C_trough_ > 20(>35) mg/L**					
<1 month	40% (10%)	X *	82% (45%)	X *	2% (0%)
1–2 months	24% (5%)	14% (2%)	35% (10%)	51% (20%)	1% (0%)
2–12 months	10% (2%)	15% (4%)	15% (2%)	38% (12%)	X *
**Efficacy: Percentage of patients with predicted 50% fT>MIC_8mg/L_ (Range: 70–100% fT>MIC_8mg/L_)**					
<1 month	100% (96–77%)	X *	100% (100–98%)	X *	99% (92–37%)
1–2 months	99% (91–62%)	95% (79–49%)	100% (96–70%)	100% (97–79%)	100% (89–37%)
2–12 months	94% (74–37%)	96% (81–46%)	98% (83–41%)	99% (98–77%)	X *

* Population was not included in model development; hence, it was not used for predictions.

## Data Availability

All demographic and simulated data are presented in the manuscript/Supplemental Materials. Further inquiries can be directed to the corresponding authors.

## Supplementary Materials

The following supporting information can be downloaded at https://www.mdpi.com/article/10.3390/pharmaceutics17050544/s1, Methods S1: Description of pharmacometric model 3 used for sensitivity analysis C. Figure S1: Flow-diagram illustrating population pharmacokinetic model selection for pharmacometric simulation studies. Figure S2: Illustration of the distribution of patient characteristics used in primary analysis; Figure S3: Comparison of predicted cefepime exposure distribution at steady state from pharmacometric simulations using model 1 and model 2, respectively. Table S1: Percentage of patients with predicted target achievement in case of high versus lower MIC (8 versus 4 mg/L) at steady-state; Data S1: Pharmacometric simulation code example.

## Abbreviations

The following abbreviations are used in this manuscript:

C_trough_	Trough concentration (total concentration)
GA	Gestational age
fT>MIC_XXmg/L_	Time during which the free drug concentration (fT) remains above a defined minimal inhibitory concentration (MIC) breakpoint (XX in mg/L)
MIC	Minimum inhibitory concentration
TDM	Therapeutic drug monitoring
PMA	Postmenstrual age
PTA	Probability of target attainment in terms of a given fT>MIC
