# Supplementary material for: Risk of Potentially Neurotoxic Exposure in Infants Under High-Dose Cefepime Treatment—A Pharmacometric Simulation Study
Source: Pharmaceutics. 2025 Apr 22;17(5):544. doi: 10.3390/pharmaceutics17050544 (PMC12114997; doi:10.3390/pharmaceutics17050544)
Supplement: Supplementary file 1 [file pharmaceutics-17-00544-s001.zip › pharmaceutics-3569301-supplementary.pdf]

Supplemental material

Supplemental Methods S1

Description of pharmacometric model 3 used for sensitivity analysis C

Zhao et al. (2020) [15] describe cefepime pharmacokinetics in a chinese population of 85 neonates, using an opportunistic sampling design (100 samples in total). A one-compartment model was used to describe distribution kinetics, cefepime clearance (CL) was described as a function of body weight, serum creatinine (Scr) and postmenstrual age (PMA), volume of distribution (V) as a linear function of weight:

V (L) = 2.07 L · (weight (kg) / 3.352 kg)

CL (L/h) = 0.589 · (weight (kg) / 3.352 kg)$^{0.75}$ · (PMA (weeks) / 40 weeks)$^{1.16}$ · (Scr (µmol/L)/28.5 µmol/L)$^{0.218}$

Unexplained inter-individual was quantified to 15% in clearance (i.e. >50% smaller than in models 1 and 2), and 27% in the volume of distribution. Residual variability was 37%.

Inclusion criteria were neonates treated regularly with cefepime, with PMA <48 weeks. Exclusion criteria were use of other antibiotics, survival time less than treatment cycle and other reasons determined by the researcher as unsuitable for inclusion.

Supplemental Figure S1

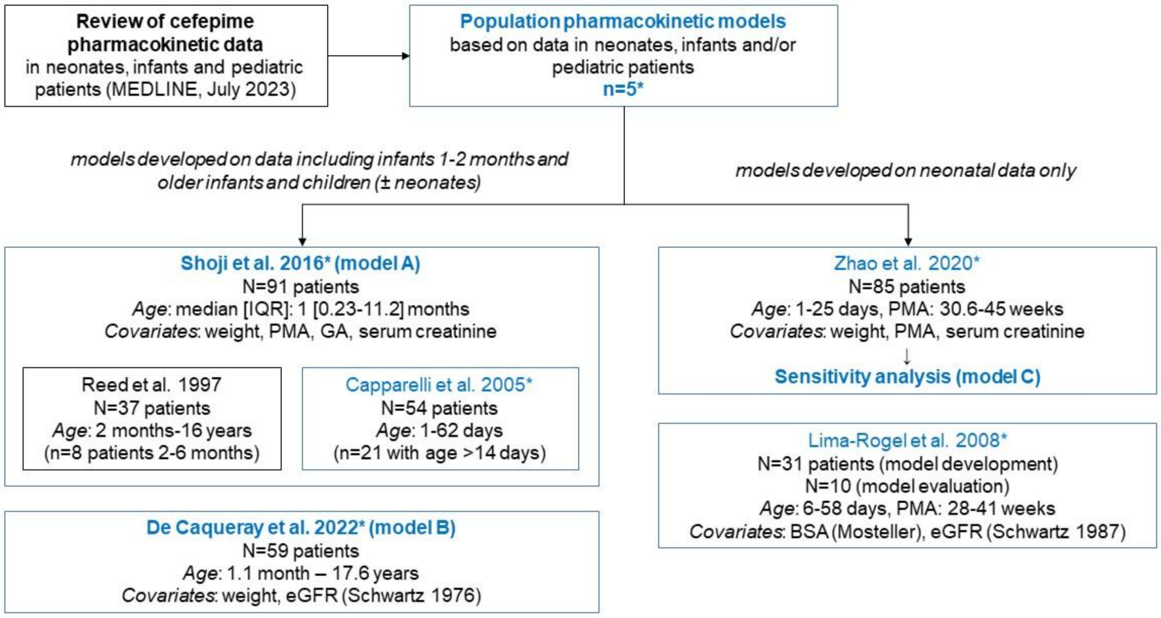

Supplemental Figure S1: Flow-diagram illustrating population pharmacokinetic model selection for pharmacometric simulation studies aiming to compare predicted exposure in infants 1-2 months of age with older infants and children (± compared with neonates). IQR: interquartile ragnge. PMA: postmenstrual age. GA: gestational age. eGFR: estimated glomerular filtration rate. BSA: body surface area.

Supplemental Figure S2

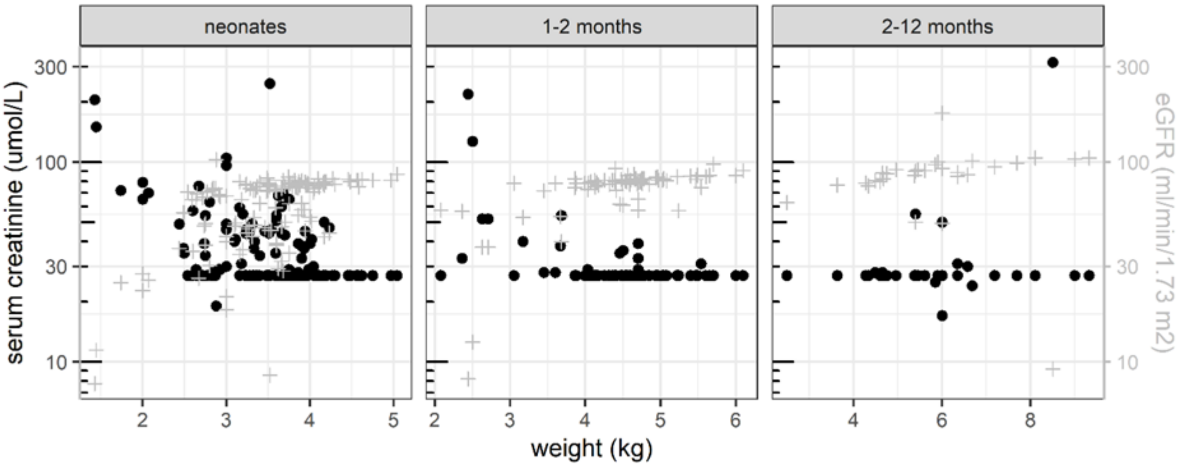

Supplemental Figure S2: Illustration of distribution of patient characteristics used in primary analysis. *Black dots*: measured serum creatinine (Jaffé method). *Grey crosses*: Estimated glomerular filtration rate (eGFR) is shown according to Schwartz (1984) [31].

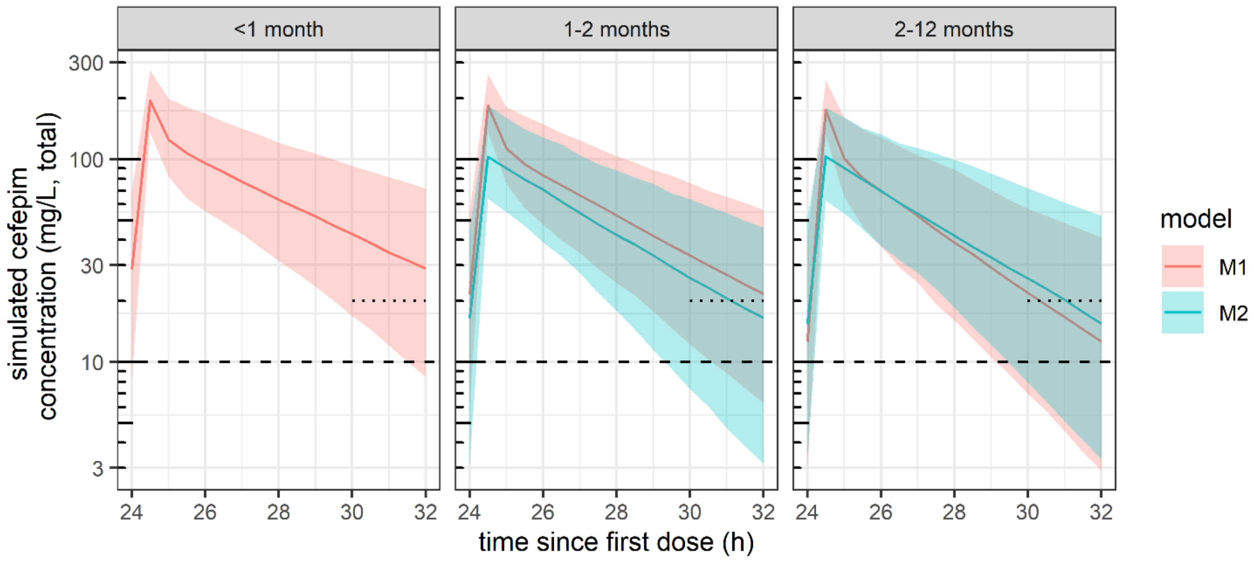

Supplemental Figure S3: Comparison of predicted cefepime exposure distribution at steady state from pharmacometric simulations using model 1 and model 2, respectively, with high-dose cefepime 50 mg/kg every 8h in all age groups. *Solid curve*: median concentration. *Shaded areas*: 90% prediction interval (5th-95th percentile). *Dotted short line*: trough concentrations >20 mg/L (evaluated lower safety threshold). *Dashed line*: Total concentration of 10 mg/L, corresponding to expected free concentration of 8 mg/L (MIC efficacy threshold evaluated).

Supplemental Table S1

Supplemental Table S1: Percentage of patients with predicted target achievement in case of high versus lower MIC (8 versus 4 mg/L) at steady-state (50 mg/kg every 8h, unless otherwise stated). Numbers indicate percentage of patients achieving 50% fT>MIC (up to 70-100% fT>MIC).

	**MIC breakpoint of 8 mg/L**		**MIC breakpoint of 4 mg/L**	
Population	Model 1	Model 2	Model 1	Model 2
<1 month	100% (99-92%)	X*	100% (100-95%)	X*
<1m, GA>36 w: 50 mg/kg every 12h	99% (85-48%)	X*	100% (98-65%)	X*
1-2 months	100% (99-85%)	99% (94-71%)	100% (100-90%)	100% (99-82%)
2-12 months	99% (92-61%)	99% (94-74%)	100% (99-73%)	100% (99-82%)

*neonates were not included in model development, hence it was not used for predictions in neonates

Supplemental Data S1: Pharmacometric simulation code example

Covariates file example

name «pop2.csv»

ID	Weight_kg	eGFR_Schwartz1976	Age_days
1	3.86	100.95	35
2	5.7	118.98	58
3	4.85	94.64	36
…			

Model file example

name «mod001.txt»

```
DESCRIPTION:
One-compartment distribution model according to de Cacqueray et al.

[COVARIATE]
input = {weight_kg, eGFR_Schwartz1976, Age_days}

; reported model parameters (population estimates with inter-individual variability
expressed as standard deviation sd) and indivdiual covariates

[INDIVIDUAL]
input = {Clpop, Vpop, sdCl, sdV, corr_Cl_V, weight_kg, eGFR_Schwartz1976, Age_days,
theGFR}

EQUATION:
Cltyp = Clpop * (weight_kg/9)^0.75 * (eGFR_Schwartz1976/153)^theGFR
Vtyp = Vpop * (weight_kg/9)

; inter-individual variability in clearance (Cl) and volume of distribution (V)
DEFINITION:
Cl = {distribution=lognormal, typical=Cltyp, sd=sdCl}
V = {distribution=lognormal, typical=Vtyp, sd=sdV}

correlation = {level=id, r(Cl, V)=corr_Cl_V}

[LONGITUDINAL]
input = {Cl, V, b}

; PK model definition and plasma concentration prediction (Cc) using a one-compartmental
model (Cl: clearance, V: volume of distribution)
Cc = pkmodel(V, Cl)

; calculation of fraction of time above threshold of 8 mg/L after 24h of dosing
; 80% free fraction (= 0.8) assumed
Threshold8 = 8
x8free = 0
if (t >= 24 & Cc*0.8 > Threshold8) x8free = 1
end

; time above threshold (free concentration)
Tabove8free_0 = 0
ddt_Tabove8free = x8free

; fraction for 8h dosing interval
fTabove8 = Tabove8/8
fTabove8free = Tabove8free/8

; proportional error model
DEFINITION:
y = {distribution = normal, prediction = Cc, errorModel=proportional(b)}
```

```
OUTPUT:
output = {Cc, x8free}
table = {Tabove8free, fTabove8free}
```

Simulx project file example

```
<MODEL>
file = 'mod001.txt'

<SIMULX>

[SETTINGS]
GLOBAL:
exportpath='sim001'

[DEFINITION]
POPULATION:
parameters={Clpop, Vpop, sdCl, sdV, corr_Cl_V, theGFR, b}
'PopParameters' = {{{values={1.21, 4.8, 0.39, 0.35, 0.5, 0.37, 0.39}}}}

OUTPUT:
'regularY_32h' = {output=y, {{start=0, interval=0.5, final=32}}}
'regularCc_32h' = {output=Cc, {{start=0, interval=0.5, final=32}}}
'regularX8free' = {output=x8free, {{start=0, interval=1, final=32}}}
'regularTabove8free' = {output=Tabove8free, {{start=0, interval=1, final=32}}}
'regularFTabove8free' = {output=fTabove8free, {{start=0, interval=1, final=32}}}

INDIVIDUAL:
parameters={Cl, V}
'IndivParameters' = {{{values={1, 1}}}}

COVARIATE:
continuousCovariates={weight_kg, eGFR_Schwartz1976, Age_days}
'Covariates' = {{{values={1, 1, 1, 1}}}}
'pop1' = {file='../Data/pop1.csv'}
'pop2' = {file='../Data/pop2.csv'}

TREATMENT:
'50mgkg_8h_inf30min' = {{{times={0, 8, 16, 24}, amounts={50, 50, 50, 50}, durations={0.5,
0.5, 0.5, 0.5}}}, adm=1, scale={rate, covariate=weight_kg, intercept=0}}

[SIMULATION]
GROUPS:
'pop1_inf30min'={size=500, parameter='PopParameters', remaining={a=1, b=1},
covariate='pop1', outputs={'regularCc_32h', 'regularFTabove8free'},
treatment={'50mgkg_8h_inf30min'}}
'pop2_inf30min'={size=500, parameter='PopParameters', remaining={a=1, b=1},
covariate='pop2', outputs={'regularCc_32h', 'regularFTabove8free'},
treatment={'50mgkg_8h_inf30min'}}

[EXPLORATION]
GROUPS:
'explorationGroup1'={remaining={a=1, b=1}, parameter='IndivParameters',
outputs={'regularCc_32h'}}

[ENDPOINT]
OUTCOME:
'Cmin_ipred_HT20_32h' = {outputElement='regularCc_32h', statistic=last, sample=value,
threshold='value>20'}
'Cmin_ipred_HT35_32h' = {outputElement='regularCc_32h', statistic=last, sample=value,
threshold='value>35'}
'fTabove8freeGT70' = {outputElement='regularFTabove8free', statistic=last, sample=value,
threshold='value>0.7'}
'fTabove8freeGT50' = {outputElement='regularFTabove8free', statistic=last, sample=value,
threshold='value>0.5'}
```

```
'fTabove8freeGT100' = {outputElement='regularFTabove8free', statistic=last, sample=value,
threshold='value>=1'}

OUTPUT:
referenceGroup='pop1_inf30min'
decisionCriterion=false
'precentHT20_32h' = { outcome='Cmin_ipred_HT20_32h', function=percentTrue,
criterion={type=statisticalTest, test='value!=1', pValue=0.05} }
'percentHT35_32h' = { outcome='Cmin_ipred_HT35_32h', function=percentTrue,
criterion={type=statisticalTest, test='value!=1', pValue=0.05} }
'percent_free_GT8_f70' = { outcome='fTabove8freeGT70', function=percentTrue,
criterion={type=statisticalTest, test='value!=1', pValue=0.05} }
'percent_free_GT8_f50' = { outcome='fTabove8freeGT50', function=percentTrue,
criterion={type=statisticalTest, test='value!=1', pValue=0.05} }
'percent_free_GT8_f100' = { outcome='fTabove8freeGT100', function=percentTrue,
criterion={type=statisticalTest, test='value!=1', pValue=0.05} }

[TASKS]
simulation()
endpoint()
```
